# Severe pre-operative sinus bradycardia improved by mediastinal lymph node dissection

**DOI:** 10.1186/s12893-022-01547-6

**Published:** 2022-03-22

**Authors:** Jin Wei, Ling Yu, Nan Wu, Hongyu Tan

**Affiliations:** 1 Department of Anesthesiology, Key laboratory of Carcinogenesis and Translational Research (Ministry of Education/Beijing), Peking University Cancer Hospital & Institute, Beijing, 100142 China; 2 Department of Thoracic Surgery II, Key laboratory of Carcinogenesis and Translational Research (Ministry of Education/Beijing), Peking University Cancer Hospital & Institute, Beijing, 100142 China

**Keywords:** Lung cancer, Severe sinus bradycardia, Mediastinal lymph node dissection, Vagus nerve

## Abstract

**Background:**

We report a case of bradycardia improved by surgical resection of the paratracheal lymph nodes, which has rarely been reported in the literature.

**Case presentation:**

A 41-year-old male patient with pre-operative sinus bradycardia was diagnosed with right upper lobe adenocarcinoma. He planned to undergo VATS right upper lobectomy and mediastinal lymph node dissection. Consultation indicated that there was no need to place a temporary pacemaker. Severe sinus bradycardia occurred during induction of anesthesia and heart rate (HR) fell significantly from 52 to 28 bpm. There was no response to atropine. Isoproterenol was administered continuously for two hours at 0.01 µg per kg per minute to keep the patient’s HR around 50 bpm. During the operation, it was noted that the right upper mediastinal lymph nodes (group R2 and R4) were calcified and very close to the vagus nerve. After resection of the lymph nodes, the patient’s HR returned to 60–68 bpm without isoproterenol. There were no post-operative complications and the patient was discharged on the 5th post-operative day. The pathological findings indicated invasive adenocarcinoma with no lymph node metastases. One month after surgery, 24-h Holter monitoring revealed sinus rhythm without bradycardia. Six months after surgery no sinus bradycardia has occurred thus far.

**Conclusions:**

Patients with persistent sinus bradycardia pre-operation caused by vagus nerve compression deserve attention. Guidelines on placement of temporary pacemakers and intraoperative anesthesia management may be improved by additional clinical experience.

## Background

Sinus bradycardia is a commonly observed arrhythmia that is usually associated with the vagally-mediated reflex in thoracic surgery. Improvement of severe sinus bradycardia following mediastinal lymph node dissection has not been previously reported. We herein present a case of a patient with severe pre-operative sinus bradycardia who underwent video-assisted thoracoscopic surgery (VATS) lobectomy of the right upper lobe with mediastinal lymph node dissection.

This report adheres to the CARE guidelines for case reports. The patient consented to the publication of this report.

## Case presentation

A 41-year-old male patient was diagnosed with lung adenocarcinoma in the right upper lobe and planned to undergo VATS right upper lobe lobectomy and mediastinal lymph node dissection. He was previously diagnosed with hypertension, which was controlled by 2.5 mg qd felodipine, and had a five-year history of cervical spondylosis. He had never taken beta blockers. The patient was a non-athlete and had no family history of malignant tumors. Pre-operative laboratory tests included full blood cell count, blood biochemistry, coagulation tests, myocardial enzyme, and tumor markers, and were unremarkable except for a slightly elevated triglyceride (2.54 mmol/L) and neuron specific enolase (19.65 ng/mL). Chest CT and PET-CT showed a 21 × 18 mm nodule in the right upper lobe with a maximum standard uptake value (SUVmax) of 4.7. There was no enlargement of mediastinal or hilar lymph nodes and cranial CT showed no clear signs of metastasis.

Notably, the patient said that his heart rate (HR) was normally around 50 beats/min (bpm) and his electrocardiogram (ECG) showed sinus bradycardia with an HR of 44 bpm. Ambulatory ECG monitoring for 24 h showed a sinus rhythm with an average HR of 42 bpm, a minimum HR of 38 bpm, and without arrest intervals greater than 2 s. The patient had no symptoms of syncope or amaurosis fugax. The intensivist was consulted and advised that there was no need to place a temporary pacemaker.

Standard monitoring on the day of the operation included measurement of the patient’s arterial blood pressure (ABP), HR, pulse oxygen saturation (SpO2), electrocardiography, and bispectral index (BIS). The patient remained in stable condition with a HR of 52 bpm, ABP of 155/61 mmHg, and SpO2 of 97% on room air. Blood gas analysis was unremarkable except for slightly elevated lactic acid of 1.9mmol/L.

**Fig. 1:**
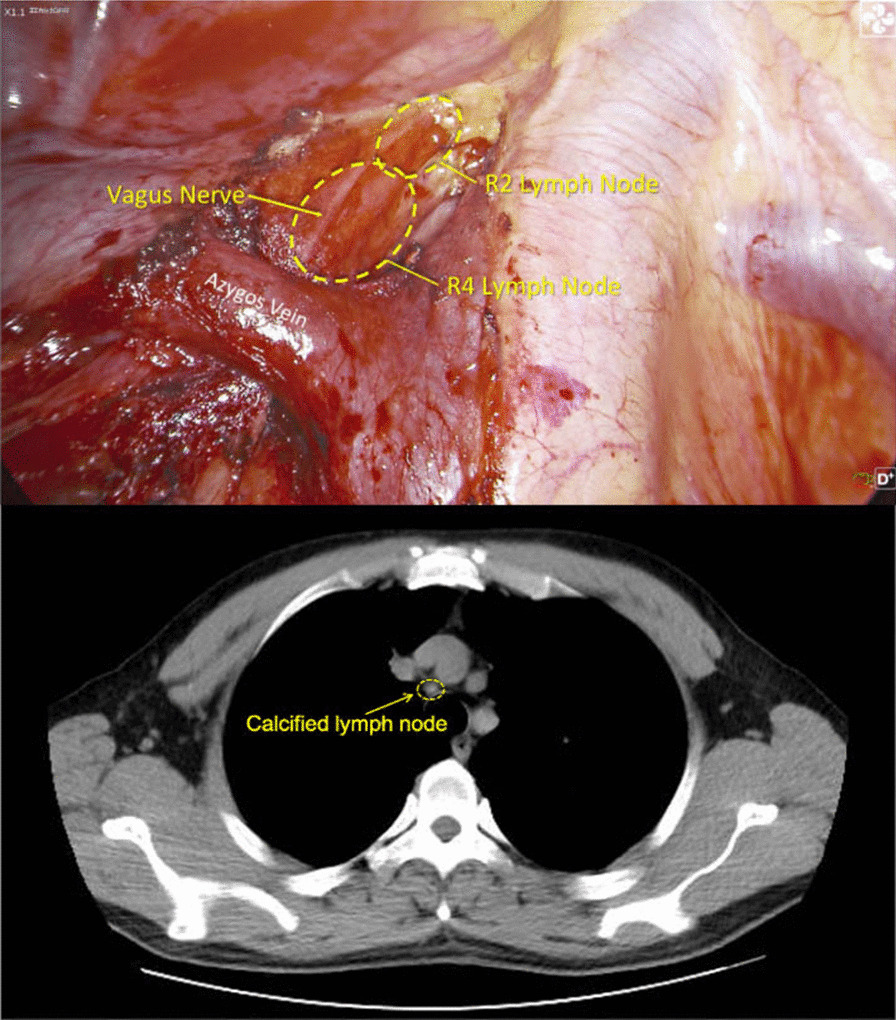
Anatomic structure of R2R4 lymph node and vagus nerve in ct-scan and surgery

Anesthesia was induced by sufentanil, propofol, etomidate, and cisatracurium. Just after induction of anesthesia severe sinus bradycardia occurred and the patient’s HR fell significantly from 52 to 28 bpm. The patient was immediately intubated with a double-lumen endotracheal tube guided by visual laryngoscope. The patient’s HR remained at 35–48 bpm and 0.5 mg of atropine was administered intravenously with no response. Isoproterenol was administered continuously for two hours at 0.01 µg per kg per minute to keep HR around 50 bpm. The patient’s ABP was 100–112/60–75 mmHg. Anesthesia was maintained by propofol, remifentanil, sevoflurane and cisatracurium. Infusion heating and a forced air warmer were used to preserve the body temperature above 36℃. During the operation, it was noted that the right upper mediastinal lymph nodes (groups R2 and R4) were calcified and very close to the vagus nerve (Fig. 1). Following resection of lymph nodes by ultrasonic knife, the patient’s HR increased to 60–68 bpm and ABP was about 130–145/80–90 mmHg. The anesthesiologist stopped isoproterenol, but HR and ABP did not decrease. We suggest that compression of the vagus nerve by the mediastinal lymph nodes may have contributed to bradycardia. The patient exhibited no postoperative complications and was discharged on post-operative day 5. The pathological findings were invasive adenocarcinoma of 3.0*2.0*1.5 cm without lymph node metastases. The stage was pT1cN0M0 (Stage 1A3). There was no post-operative adjuvant treatment plan for this patient.

Based on these conditions, we requested that the patient repeat 24-h Holter monitoring. One month after surgery, 24-h Holter monitoring revealed a sinus rhythm with an average heart rate of 67 bpm and without intervals of more than 2 s. Six months after surgery no sinus bradycardia has occurred thus far and the patient has reported that his heart rate is now 60–80 bpm normally.

## Discussion and conclusions

The immediate relief of bradycardia following lymph node resection is an unexpected surprise to the anesthesiologist. Systematic literature searches for keywords “lobectomy” and “preoperative sinus bradycardia” in Pubmed, Embase, CNKI, and Chinese Wanfang databases identified only one brief report in Chinese Wanfang databases from nearly ten years ago. Literature search for keywords “lobectomy”and ”vagus nerve” revealed fourteen articles, but they were primarily about transient bradycardia caused by intraoperative traction. Keyword search for ”bradycardia” and ”vagus nerve” expanded these literature findings to nearly eighty articles, but did not identify any reports of recovery from bradycardia after lymphadenectomy.

Severe sinus bradycardia during surgery is a great challenge for anesthesia management, as it is associated with reduced cardiac reserve and increased risk of progression to cardiac arrest [1–3]. Known factors that may cause sinus bradycardia are drug influence, electrolyte disorder, hypothyroidism, intracranial hypertension, acute myocardial infarction, hypothermia, sick sinus syndrome and high parasympathetic nerve tension.

The vagus nerve is a mixed nerve that innervates the secretion of myocardium, smooth muscle, and glands. It is critical for maintenance of normal physiological activities and for functional regulation of the cardiovascular system and internal organs. Branches of the vagus nerve are widely distributed throughout the trachea, bronchus, and esophagus, and form the pulmonary plexus in the hilum of the lung. Tension or “pulling” on the vagus nerve between the hilum and esophagus may increase parasympathetic output and lead to reflex bradycardia. Reducing this pulling action or surgical relief of any local vagus nerve block can significantly reduce this reflex.

**Fig. 2:**
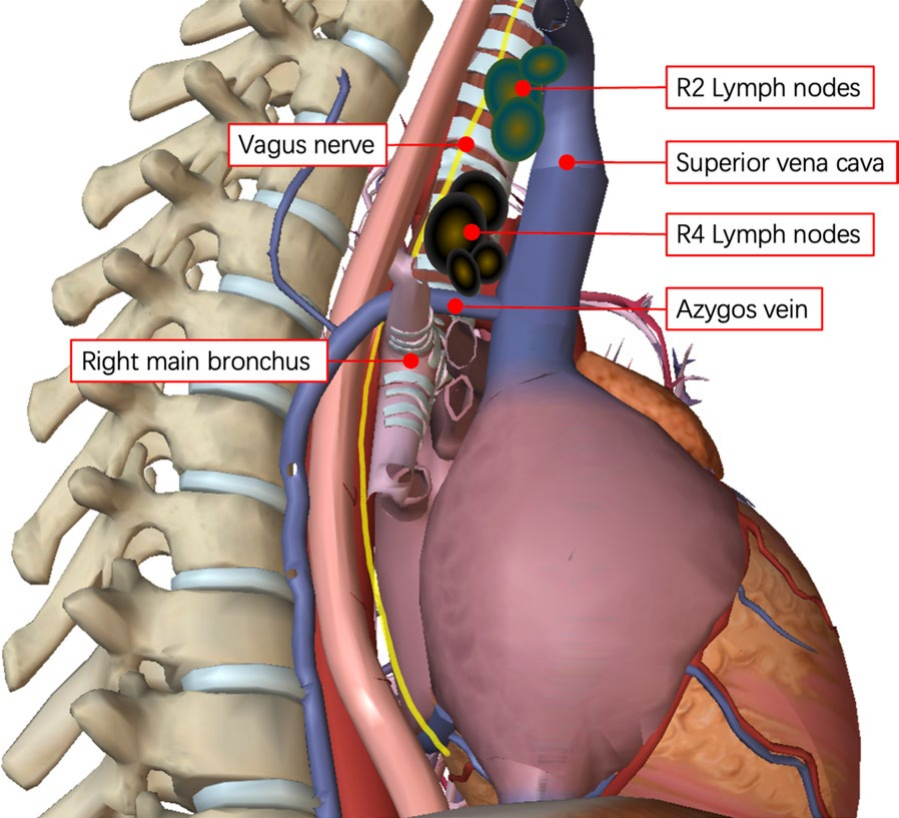
Detailed anatomical sketch of the operative situs with nerve and lymph node stations

In this case, the patient had severe sinus bradycardia before operation that was significantly improved after dissection of the R2 and R4 lymph nodes. Comparison of 24-hour Holter monitoring data acquired pre- and post-operation showed that the patient’s average HR increased from 42 bpm to 67 bpm, respectively. The lymph nodes of group R2 and R4 are located between the trachea, superior vena cava and azygos vein, close to the vagus nerve (Fig. 2). Bradycardia observed during lung cancer operations is assumed to be due to surgical stimulation of the vagal nerve that initiates the reflex. The afferent limb of the reflex involves the pulmonary plexus of the vagal nerve, and the efferent limb involves the cardiac vagus nerve [1, 2, 4]. Reflex bradycardia after a specific surgical maneuver is a phenomenon well known to surgeons. Most cases of bradycardia occur because of vagally mediated reflection after stretching of the vagus nerve. In these situations, reflex bradycardia usually resolves after ceasing the causative surgical maneuver or following administration of intravenous anticholinergics (atropine) and/or β Receptor agonists (Isoproterenol). Anticholinergics and β Receptor agonists play an important role in positive timing and positive conduction by regulating the autonomic nervous system. They are common drugs for the treatment of acute bradycardia.

In this case, severe sinus bradycardia occurred before surgery, and was further aggravated after induction of anesthesia. In order to treat severe sinus bradycardia, the isoproterenol was used during surgery, and we maintained the heart rate at around 50 bpm. No obvious HR change was found by surgical stimulation, which may be related to the usage of isoproterenol. We think the severe sinus bradycardia was due to the persistent mechanical stimulation of the vagal nerve caused by the compression of lymph nodes.

The persistent effect of lymph nodes compression on the vagus nerve is rare in a lobectomy. We believe that the observed pre-operative sinus bradycardia in this case is likely attributable to increased vagal tone caused by the constriction of the vagus nerve between the calcified R2/R4 lymph nodes. The severe sinus bradycardia was further aggravated after induction of anesthesia and relieved after lymph node removal. Aggravation of bradycardia after induction may be related to the application of muscle relaxants, in this case cisatracurium, which relax the skeletal muscle and may increase constriction of the vagus nerve by the calcified lymph nodes. In addition, the use of sufentanil may further influence the occurrence of sinus bradycardia. After mediastinal lymph node dissection, the constriction was relieved, vagus nerve tension returned to normal, and the patient’s heart rate recovered spontaneously.

This patient had a good prognosis, however, some points should be considered. First, the necessity of installing temporary pacemakers may require further analysis. According to the 2018 ACC/AHA/HRS guidelines [5], sinus node dysfunction is defined as both sinus bradycardia (heart rate < 50 bpm) and sinus arrest time greater than 3 s. The treatment of sinoatrial node dysfunction mainly depends on whether there are clinical symptoms or the operations are easy to cause bradycardia. In our case, the most common arrhythmia is atrial fibrillation with an incidence of 10–40% for all pulmonary resections, and 33% for lobectomy [6]. The lobectomy is not likely to cause bradycardiac arrhythmia, and this patient had no symptoms of syncope or amaurosis fugax, so temporary pacemaker implantation was not deemed necessary after consulation by an intensivist. In this instance further evaluation by professional cardiologists may have prevented the severe sinus bradycardia that occurred during the operation.

Secondly, the patient’s chest CT was reevaluated with the surgeon and an experienced radiologist post-operation. We found that the calcification of right upper mediastinal lymph nodes (group R2 and R4) and their proximity to the vagus nerve was detectable in the pre-operation CT images. However, because this had no impact on the surgical technique, it did not attract attention before the operation. According to this case, anesthesiologists should not ignore enlargement or calcification of lymph nodes near the vagus nerve.

Patients with persistent sinus bradycardia pre-operation caused by vagus nerve compression deserve attention. More clinical experience in this area may improve intraoperative anesthesia management and guidelines for the use of temporary pacemakers.

## Data Availability

The datasets used during the current study are available from the corresponding author on reasonable request.

## Abbreviations

ECG: Electrocardiogram
ABP: Arterial blood pressure
HR: Heart rate
SpO2: Pulse oxygen saturation
BIS: Bispectral index
Bpm: Beats/min
CT: Computer Tomography
PET-CT: Positron Emission Tomography-Computer Tomography
SUVmax: Maximum standard uptake value
